# The Expression of NOX From Synthetic Promoters Reveals an Important Role of the Redox Status in Regulating Secondary Metabolism of *Saccharopolyspora erythraea*

**DOI:** 10.3389/fbioe.2020.00818

**Published:** 2020-07-17

**Authors:** Xiaobo Li, Ju Chu, Peter R. Jensen

**Affiliations:** ^1^State Key Laboratory of Bioreactor Engineering, East China University of Science and Technology, Shanghai, China; ^2^National Food Institute, Technical University of Denmark, Kongens Lyngby, Denmark

**Keywords:** synthetic promoters, redox regulation, NADH oxidase, *Saccharopolyspora erythraea*, secondary metabolism, c-di-GMP

## Abstract

Redox cofactors play a pivotal role in primary cellular metabolism, whereas the clear link between redox status and secondary metabolism is still vague. In this study we investigated effects of redox perturbation on the production of erythromycin in *Saccharopolyspora erythraea* by expressing the water-forming NADH oxidase (NOX) from *Streptococcus pneumonia* at different levels with synthetic promoters. The expression of NOX reduced the intracellular [NADH]/[NAD^+^] ratio significantly in *S. erythraea* which resulted in an increased production of erythromycin by 19∼29% and this increment rose to 60% as more oxygen was supplied. In contrast, the lower redox ratio resulted in a decreased production of another secondary metabolite, the reddish pigment 7-O-rahmnosyl flaviolin. The metabolic shifts of secondary metabolism results in a higher NADH availability which compensates for its oxidization via NOX. The expression of the erythromycin biosynthesis gene cluster (BGC) in the NOX-expression strains was upregulated as the activity of diguanylate cyclase was inhibited moderately by NADH. This study also suggested that lower intracellular [NADH]/[NAD^+^] ratio benefits the biosynthesis of erythromycin by potentially affecting the biosynthesis of the secondary messenger, bis-(3′-5′)-cyclic dimeric guanosine monophosphate (c-di-GMP), which may stimulate the positive regulation of erythromycin BGC via BldD. The present work provides a basis for future cofactor manipulation in *S. erythraea* to improve the industrial production of erythromycin.

## Introduction

Derivatives of currently prevalent macrolides, e.g., erythromycin, are among the most frequently used chemotherapeutic agents for treating infections. Erythromycin, which is produced industrially by *Saccharopolyspora erythraea* (*S. erythraea*), and its derivatives show antimicrobial activities against a broad-spectrum of pathogens (Mironov et al., 2004). *S. erythraea* is also a particularly interesting organism to study as it represents a model system for biosynthesis of natural products in actinomycetes (Leadlay, 1997). The biosynthesis of erythromycin is involved in a complex secondary metabolic network, that is connected with the primary metabolism via specific precursors and cofactors, including a supply of NADPH as reducing equivalents (McPherson et al., 1998). However, knowledge about effects of other cofactor metabolism in perturbation on *S. erythraea* is scarce. Like other secondary metabolites, the biosynthesis of erythromycin is linked to signal transduction and transcriptional regulation. The transcription of the erythromycin biosynthesis gene cluster (BGC) is under the positive regulation by BldD, which can bind all the promoter regions in the BGC (Chng et al., 2008). It has been proven previously that the nucleotide-based secondary messenger, bis-(3′-5′)-cyclic dimeric guanosine monophosphate (c-di-GMP), even at very low concentrations activates the binding between BldD and the BGC (Tschowri et al., 2014; Xu et al., 2019). c-di-GMP allows a rapid integration of external and internal signals into fine-tuned regulatory pathways, which controls the cellular responses to changing environmental conditions (Tschowri et al., 2014) but the signals involved in the c-di-GMP-related regulatory networks are still unknown.

The cellular redox status, as reflected in the NADH/NAD^+^ ratio, is one of key signals that can influence on the cellular metabolism (Brekasis and Paget, 2003). In microorganisms NAD(H) acts as coenzymes for hundreds of redox reactions (Qin et al., 2009). The majority of NADH is produced from glycolysis and the TCA cycle, pathways from which some precursors for secondary metabolites are also generated. As such, the cellular redox status is closely linked to the secondary metabolism. For example, inhibition of three enzymes in the TCA cycle by NADH resulted in a decrease of the carbon flux toward the TCA cycle and prompted polyketides production in streptomycetes (Wang et al., 2020). On the other hand, a higher carbon flux through the TCA cycle contributed to enhance the accumulation of precursors for erythromycin (Hong et al., 2016). This apparent discrepancy stresses the complex role of the redox status beyond the central carbon metabolism, including the biosynthesis of secondary metabolites.

By regulation of enzyme activities in the central carbon metabolism, e.g., glyceraldehyde-3-phosphate dehydrogenase or lactate dehydrogenase (Ying, 2006), changes of the NAD(H) pool reroute the carbon flux (Qiao et al., 2017). Thus, in addition to two widely used strategies, i.e., the deletion/overexpression of genes encoding key enzymes (Reeves et al., 2007; Borodina et al., 2008; You et al., 2019) and engineering regulatory networks of biosynthesis pathways (Fernandez-Martinez et al., 2015; Liu et al., 2017; Horbal et al., 2018), metabolic engineering by manipulating redox cofactors has proven to be another effective strategy particularly for yield enhancement of primary metabolites derived from the central carbon metabolism (Dugar and Stephanopoulos, 2011). Furthermore, redox rebalancing is essential to restore growth and enhance production particularly in strains that are metabolically engineered (Zhang et al., 2014; Man et al., 2016; Wang et al., 2017). On the other hand, the investigation of physiological responses toward cofactor engineering through analysis at transcriptional or metabolic levels facilitates to understand intracellular biological processes. For instance, the metabolic networks controlled by NADH were identified in *Escherichia coli* and the factor to trigger Crabtree effect in *Saccharomyces cerevisiae* was disclosed by transcriptional analysis (Vemuri et al., 2007; Holm et al., 2010).

The intracellular redox status can be tuned through manipulations of cofactor availability, switching cofactor preference of key enzymes or heterologous expression of NAD(P)H-relating enzymes (Berríos-Rivera, 2002; Qiao et al., 2017). For instance, the water-forming NADH oxidase (NOX) from *Streptococcus pneumonia* (Auzat et al., 1999) has been used to change redox status in several microorganisms (Vemuri et al., 2007; Hou et al., 2009). In *S. erythraea*, there is no orthologous gene to *nox* (Oliynyk et al., 2007) so the expression of NOX from *S. pneumonia* provides a tool to manipulate intracellular redox status in this organism. Owing to the predictable drawbacks of strong perturbations of redox status (Zhang et al., 2014; Man et al., 2016), moderate redox perturbations in *S. erythraea* is preferable. This requires delicate genetic tools in *S. erythraea* to fine tune the cellular redox status by expressing the NOX moderately. The concept of synthetic promoter library (SPL) makes it possible to achieve this goal (Jensen and Hammer, 1998a; Solem and Jensen, 2002) as a library consisting of a set of promoters enable precise control of gene expression across a continuum of broad expression levels. The sequence of spacers between the consensus sequences, i.e., -35 and -10 regions of a promoter modulates the strength of prokaryotic promoters (Jensen and Hammer, 1998b). As such, a SPL can be constructed by keeping the -35 and -10 sequences almost intact and randomizing the surrounding nucleotides. In principle, once the consensus of -35 and -10 sequence of a promoter are disclosed, such a SPL containing promoters with varying strengths can be constructed on the basis of that known promoter (Hammer et al., 2006).

Our recent study showed that the biosynthesis of erythromycin was positively correlated with intracellular ATP/ADP ratio (Li et al., 2020). During fully aerobic respiration in *S. erythraea*, oxygen utilization couples NADH oxidation to ATP generation. NADH can be re-oxidized via the electron transport chain (ETC), and the proton gradient is subsequently used to drive ATP synthesis. Noteworthy, two types of terminal oxidase, i.e., *bd* and *bc_1_-aa_3_* oxidases with different energetic efficiency are used for transporting electrons in *S. erythraea* or streptomycetes (Sawers et al., 2016), which affects the yield of ATP per NADH. It is also known that the industrial production of some antibiotics are in high demand of oxygen, reflecting in the indispensability of maintaining high dissolved oxygen (DO) through the cultivation (Yegneswaran et al., 1991). However, the role priority of oxygen in the biosynthesis of antibiotics is still not fully understood, which complicates further process optimization.

The objective of the present work is to explore the possibility of improving erythromycin production by fine tuning the intracellular redox status in *S. erythraea* and elucidate the signals and mechanisms triggering the physiological response toward disturbed NADH/NAD^+^ ratio. To achieve this goal, a SPL for *S. erythraea* was constructed and NOX from *S. pneumonia* was expressed at different levels. The resulting physiological response in the NOX-expression strains was characterized, including changes of secondary metabolism and allowed us to establish a link between the cellular redox signal and transcriptional regulation of the erythromycin BGC.

## Materials and Methods

### Construction of Plasmids and Strains

Strains and plasmids used in this study were listed in Table 1. Sequences of all the primers were listed in Table 2. The fragment of *gusA* was amplified with primers gusAF2 and SPLRA using pSETGUS as the template. *gusA* was then cloned into pIB139 between *Xba*I and *Eco*RI sites yielding pIBGUS. Primers SPLF1/2/3/4 and SPLRA were used pairwise to amplify SPL-gusA fragment, which was cloned into pIB139 between *Nsi*I and *Eco*RI sites yielding pSPLGUS.

**TABLE 1:** Strains and plasmids used in this study.

**Designation**	**Genotype or description**	**References**
***S. erythraea* strains**		
E3	Industrial erythromycin producing strain	Our lab
E3:GUSA	Single copy of ermE*p*-gusA* expressed in E3	This work
D1N	Single copy of *D1W12*p-*nox* expressed in E3	This work
A2N	Single copy of *2A23*p-*nox* expressed in E3	This work
R3N	Single copy of ermE*p-*nox* expressed in E3	This work
E3H	F1F0-ATPase overexpressed in E3, promoted by its native promoter, lower [ATP]/[ADP] ratio compared to E3	Li et al., 2020
***E. coli* strains**		
Top 10	F- mcrA Δ(mrr-hsdRMS-mcrBC) φ80 lacZΔM15Δ lacX74 recA1 araΔ139Δ(ara-leu)7697 galU galKrpsL (StrR) endA1 nupG, used for plasmid constructions	
ET12567	F- dam-13:Tn9 dcm-6 hsdM hsdR zjj-202:Tn10 recF143 galK2 galT22 ara-14 lacY1 xyl-5 leuB6 thi-1 tonA31 rpsL136 hisG4 tsx-78 mtl-1 glnV44, used for conjugation	
**Plasmids**		
pIB139	An integrative plasmid containing oriT, attP, int, aac(3)IV and ermE*p	Wilkinson et al., 2002
pSETGUS	*gusA* containing *Bam*HI fragment cloned into *Bam*HI site of pSET152	Myronovskyi et al., 2011
pIBGUS	pIB139 derivative with ermE*p in front of *gusA*	This work
pSPLGUS	pIB139 derivatives with synthetic promoters in front of *gusA*	This work
pNOX	CDS of *nox* inserted into pIB139 without any promoters	This work
pD12nox	*D1W12*p-*nox* inserted into pIB139 without original ermE*p	This work
p2A23nox	*2A23*p-*nox* inserted into pIB139 without original ermE*p	This work
pIBnox	CDS of *nox* inserted into pIB139, promoted by ermE*p	This work

**TABLE 2:** Primers used in this study.

**Designation**	**Sequence (5′-3′)**
gusAF2	GGGTTTTCTAGAAGCAACGGAGGTACGGACTTGCTCCGGCCCGTCGAAACCC
SPLRA	CCGGAATTCTCACTGCTTCCCGCCCTGCTG
SPLF1	AATCCAATGCATTGGTTCTGCANNNNNNNNNNTTGANNNNNNNNNNNNNNNNNNNTNNNNTNNNNNTCTAGAAGCAACGGAGGTACGGAC
SPLF2	AATCCAATGCATTGGTTCTGCANNNNNNNNNNTGWVDVNNNNNNNNNNNNNNNNNTNNNNTNNNNNTCTAGAAGCAACGGAGGTACGGAC
SPLF3	AATCCAATGCATTGGTTCTGCANNNNNNNNNNGAAAHSNNNNNNNNNNNNNNNNNTNNNNTNNNNNTCTAGAAGCAACGGAGGTACGGAC
SPLF4	AATCCAATGCATTGGTTCTGCANNNNNNNNNNACCAAGNNNNNNNNNNNNNNNNNTNNNNTNNNNNTCTAGAAGCAACGGAGGTACGGAC
SEQF2	ATGTGCTGCAAGGCGATTAAGTTGGGT
SEQR2	AGTTCTCCCGGTCGAGGCTGAACGC
NOXF2	TTGGTAGGATCCACATATGAGCAACGGAGGTACGGACATG
NOXR	GCGCGGCCGCGGATCCTCTAGATCACTTCTCCGCCGTCAG
NOXF	AGGTCGACTCTAGTATGCATAGCAACGGAGGTACGGACATG
WPF2	GTCGACTCTAGTATGCATTGGTTCTGCA
WPR	GTCCGTACCTCCGTTGCT
WPC	GCTTCTCGTAGGACTCCTTGTG

*Restriction sites are underlined.*

The coding sequence (CDS) of *nox* from *S. pneumonia* was codon optimized for *S. erythraea* by Integrated DNA Technologies, Inc. (United States) according to codon preference in *Streptomyces coelicolor*, as *S. erythraea* and *S. coelicolor* show high genomic similarity (Oliynyk et al., 2007). Primers NOXF2 and NOXR were used for amplification of CDS of *nox*, which was subsequently inserted into pIB139 between *Nde*I and *Xba*I yielding pIBnox. Primers NOXF and NOXR were also used for amplification of *nox*, which was subsequently inserted into pIB139 between *Nsi*I and *Xba*I yielding pNOX. Synthetic promoters were amplified with primers WPF2 and WPR, and then were cloned into pNOX at *Nsi*I yielding pD12nox and p2A23nox, respectively. Primers SEQF2 and WPC were for PCR confirmation of NOX-expression strains of *S. erythraea*.

Plasmids were transformed into *E. coli* ET12567 for demethylation and next were introduced into *S. erythraea* E3 through transconjugation on ISP4 agar medium (Hopwood et al., 1985). 30 μg apramycin was overlaid on ISP4 plates for screening exconjugants, followed by PCR identification.

*S. erythraea* was cultivated on XM agar medium (Chen et al., 2017) at 34°C for 6 days to collect spores. About 1 cm^2^ XM agar medium covered with dense spores was picked into flasks with preculture medium (Chen et al., 2017). The preculture was shaken with 220 rpm at 34°C. After the 48 h preculture, 3 mL preculture was collected, resuspended in 3 mL PBS buffer (pH = 7.4) and inoculated into 300 mL flasks with either 27 mL modified minimal liquid medium for physiological study or 27 mL complex fermentation medium (Chen et al., 2017) for characterization under industrial condition. The modified minimal liquid medium (MMLM) contained the following per 800 mL: 2 g (NH_4_)_2_SO_4_, 5 g casamino acids (Difco^TM^, BD), 0.6 g MgSO_4_.7H_2_O, 0.001 g ZnSO_4_.7H_2_O, 0.001 g FeSO_4_.7H_2_O, 0.001 g MnCl_2_.4H_2_O, 0.001 g CaCl_2_. 800 mL MMLM was then dispensed in 80 ml aliquots. 15 mL NaH_2_PO_4_/K_2_HPO_4_ buffer (0.1 M, pH = 6.8) was added in 80 mL liquid medium for pH buffering. After autoclaving, 25% (w/v) glucose as sole carbon source was injected in the medium to a final concentration 20 g/L. Liquid culture was performed in shaking water baths (Julabo, Germany) with an agitation speed of 140 rpm at 34°C.

To elucidate the oxygen effects both on the control strain E3 and NOX-expression strains, another two types of 250 mL shake flasks (Schott, Germany) were used. The bottleneck diameter of type A and B flask is 3.8 and 5.6 cm, which contained 30 mL and 15 mL final volume of minimal liquid medium, respectively.

### SPL Strength Measurement and Sequencing

One milliliter liquid trypticase soy broth (TSB) with 500 μg/mL 5-bromo-4-chloro-3-indolyl-β-D-glucuronide (X-Gluc) was overlaid on the ISP4 plates with SPL exconjugants. After overnight incubation at 34°C, single colonies with blue halos were transferred to a new XM plate. After incubation at 34°C for another 6 days, single colonies were picked into 100 mL flasks with 10 mL preculture medium. 3 mL preculture was harvested at 48 h and transferred to 27 mL modified minimal liquid medium in 300 mL flasks. 1 mL culture was used for the determination of glucuronidase activity in cell lysates, which was described previously (Siegl et al., 2013).

Mycelia of single colonies with synthetic promoters were scraped from the XM plates and transferred to a microtube with 20 μL H_2_O. The tubes were boiled for 15 min at 100°C. After this step, the solution was spun down at top speed for 10 s, 1.5 μL of the supernatant was used as the PCR template in a 20 μL reaction. Primers SEQF2 and SEQR2 were used for normal sequencing of promoters.

### Fermentation Analysis

Cell samples from cultures in triplicate shake flask were collected over the time course. Dry cell weight (DCW) in 3 mL culture was measured for monitoring cell growth (Borodina et al., 2008). Concentration of residual glucose in culture was determined by salicylic acid method (Zou et al., 2009b). The DO concentration was determined using a DO electrode (Endress+Hauser A/S, Denmark). 25 mL broth with cells was withdrawn from flasks for oxygen determination. Then DO was monitored each 10 s in a total duration of 5 min using the same probe for all samples. The erythromycin titer was measured by the modified H_2_SO_4_-colorimetric method (Zou et al., 2009a).

### Analysis of Intracellular Cofactors

For [NADH]/[NAD^+^] determination, cold methanol quenching was applied. Previous reports on actinomycetes suggest leakage caused by cold shock (Wittmann et al., 2004). Each sample with 50 μL cell culture was quenched by immediate addition of 500 μL methanol precooled at −80°C. 500 μL 4°C water and 500 μL −20°C chloroform were instantly added to samples. Samples was vortex vigorously and then stored at −20°C for 1 h. Supernatant was reserved by centrifugation at 10000 × *g* at 4°C for 10 min. NAD/NADH-Glo^TM^ assay kit (Promega, United States) was used for NADH/NAD^+^ measurement. For [ATP]/[ADP] ratio determination, cells were quenched by phenol as described previously (Michel et al., 2015). The amount of ATP or ADP was quantified with ATP determination Kit (A22066) (Invitrogen, United States). ADP was assayed after ATP had been determined by adding pyruvate kinase and recording the increase in luminescence. The results were corrected for quenching of the signal by the addition of pyruvate kinase.

The *P* value of their significance was calculated using Student’s *t* test.

### Next-Generation Sequencing of RNA

Two replicates from independent cultures in modified minimal liquid cultures were used. Cells were harvested at 4 h in the early exponential phase for total RNA extraction. 10 mL culture was centrifuged at 4000 × *g* at 4°C for 10 min. The supernatant was discarded and the cells were resuspended in 2 mL RNAlater^TM^ solution (Invitrogen, United States). Total RNA was extracted with an RNeasy Plus Mini kit (Qiagen, Germany) using glass beads to mechanically disrupting cells with a FastPrep. DNase treatment by RNase-Free DNase Set (Qiagen, Germany) aided to digest DNA in samples. The RNA integrality was analyzed with 1% agarose gel electrophoresis and Bioanalyzer (Agilent, United States). The RNA concentration was determined with Bioanalyzer (Agilent, United States). RNA sequencing was accomplished using BGIseq 500 next-gen sequencer by BGI, China. Datasets consisted of at least 30 M reads per sample. After sequencing, the raw reads were filtered by removing adaptor sequences, contamination and low-quality reads from raw reads.

### Transcriptomic Analysis

FastQ files sequenced by BGIseq 500 were input into the software, Geneious Prime, for raw reads mapping into *S. erythraea* E3 genome (Li et al., 2013) and were trimmed afterward. Geneious Prime was used for calculation of RPKM value of each gene and visualization of transcripts. Differentially expressed genes (DEGs) were identified by using the DEseq2 method with criteria that are both log2 (fold change) >0.75 and adjusted *P* values < 0.01 for each gene. Clusters of orthologous group were identified in EggNOG database (Huerta-Cepas et al., 2016).

### *In vitro* Enzymatic Assays

One milliliter culture from batch cultures in shake flasks was harvested at the end of exponential phase. Cell crude extracts were prepared from the pellets with a bead beater (FastPrep), which shaked vigorously for 30 s. Repeat the shaking for four times. The NOX reaction buffer contains 50 mM potassium phosphate buffer (pH 7.0), 0.4 mM NADH, and 0.3 mM EDTA. The reaction was initiated by the addition of a suitable amount of extract (0.5–5 ml) and monitored by the absorbance decrease in A_340_. A unit of enzyme was defined as the amount which catalyzed the oxidation of 1 μmol of NADH to NAD per minute at 25°C.

Ten milliliter culture containing E3 in minimal liquid medium was harvested at 16 h and pelleted, followed by resuspension in 3 mL protein lysis buffer which containing Tris–HCl (pH = 8.0) 20 mM and protease inhibitor cocktail 5% (v/v). The cells were grinded with liquid nitrogen immediately after collection, which was followed by extensive centrifugation, 5000 rpm for 10 min at 4°C. Protein concentration was determined by Bradford assay (Bradford, 1976). The standard assay mixture contained: 50 mM pH 7.5 Tris–HCl buffer, MgCl_2_ 10 mM, EDTA 1 mM, and 0.2 mM GTP. 0.1 mL standard assay mixture was used for catalysis reaction of diguanylate cyclase. The reaction was initiated by adding the whole cell extract containing approximately 10 μg protein. Following incubation at 30°C for 20 min, the reaction was terminated by heating at 100°C for 3 min. The enzymatic activity of diguanylate cyclase was determined by measuring formation of c-di-GMP with ELISA kit (MyBioSource, United States). The standard curve of c-di-GMP was plotted by non-linear curve fit (allometric1) with the software OriginPro 2018b.

## Results

### Construction of a Synthetic Promoter Library for Fine-Tuning of Intracellular Redox Status in *S. erythraea*

**FIGURE 1:**
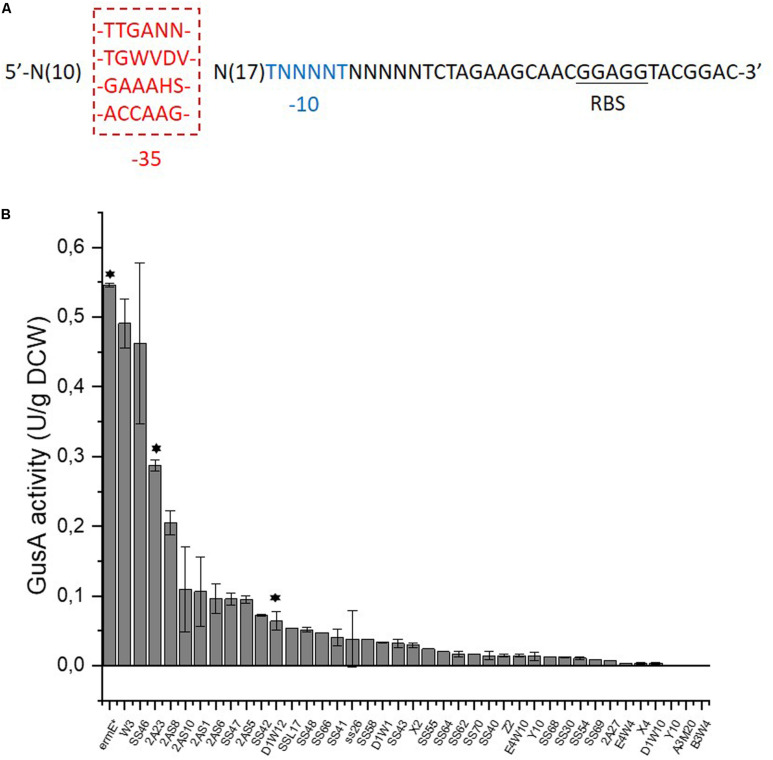
Construction of a synthetic promoter library in *S. erythraea*. **(A)**, four sets of templates for the construction of a SPL. -35 or -10 region is in red or blue, respectively. Ribosome binding site (RBS) is underlined. The degenerate nucleotides: N = A/C/G/T, W = A/T, V = A/C/G, D = A/G/T, H = A/C/T, S = C/G. **(B)**, characterization of promoter strength shown as the total GusA activity per gram of dry cell weight (DCW). Asterisks above bars indicate that these promoters would be used to drive the expression of NOX.

In order to achieve fine tuning of redox status, we developed promoters with various strengths to precisely control the gene expression, using the concept of a SPL which should meet this demand (Jensen and Hammer, 1998a). As a starting point for constructing a SPL, the strong promoters of 16s rRNA genes (SACE_8101/8105/8112/8116) were employed as the starting point to construct a SPL in *S. erythraea* (Oliynyk et al., 2007). The transcription level of the rRNA genes are almost twice that of the widely used strong constitutive promoter *ermE*^∗^p (Wilkinson et al., 2002). As there is little information about the exact promoter regions of the 16s rRNA genes in *S. erythraea* and furthermore promoters in *S. erythraea* and streptomycetes generally show a wide diversity in sequences (Strohl, 1992), we tried a simple and fast method to identify the putative promoter regions of the 16s rRNA genes in *S. erythraea*. We summarized the consensus sequences of all 16s rRNA promoter regions in another model strain *S. coelicolor*, from where two consensus sequence combinations were previously extracted (Baylis and Bibb, 1988; van Wezel et al., 1994; Supplementary Figure S1). Due to the high similarity between genomes of *S. erythraea* and *S. coelicolor* (Oliynyk et al., 2007), the two consensus sequences from *S. coelicolor* were aligned to the upstream regions of the 16s rRNA genes to identify putative promoters in *S. erythraea*. From the resulting alignments four sets of consensus sequences were derived, which then served as templates to synthesize promoters in this study (Figure 1A and Supplementary Figure S1). The spacer sequence between the -35 and -10 hexamers was entirely randomized, so was the ten nucleotides upstream of the -35 region and the five nucleotides downstream of the -10 region. The length of the spacer region was fixed to 17 nucleotides as it is the average length of the spacer region of σ^70^-like promoters in streptomycetes (Strohl, 1992).

The heterologous *gusA* encoding β-glucuronidase (Myronovskyi et al., 2011), was employed in *S. erythraea* as the reporter gene for synthetic promoters. A promoter library with 39 promoters was constructed and is shown in Figure 1B and Supplementary Table S1. Activities of these promoters range from 0.1 to 90% strength of the strong constitutive *ermE*^∗^p. All the four templates used provided functioning promoters in *S. erythraea* and demonstrates that this simplified strategy allowed us to conveniently expand the utility of the SPL concept for *S. erythraea*.

### Differential Expression of NOX in *S. erythraea*

The water-forming NOX from *S. pneumoniae* were expressed in *S. erythraea* to disturb the intracellular redox status. Prior to its expression, the CDS of *nox* was codon optimized (Supplementary Figure S2). This strategy is expected to invoke an unbiased response, in contrast to deleting or overexpressing metabolic pathways directly coupled to cofactor utilization, which may result in localized changes in fluxes surrounding the reaction. A high erythromycin-producing strain *S. erythraea* E3 obtained from random mutagenesis and screening (Li et al., 2013) was employed to test whether redox regulation could be a feasible and straightforward strategy to improve the industrial production of erythromycin.

**FIGURE 2:**
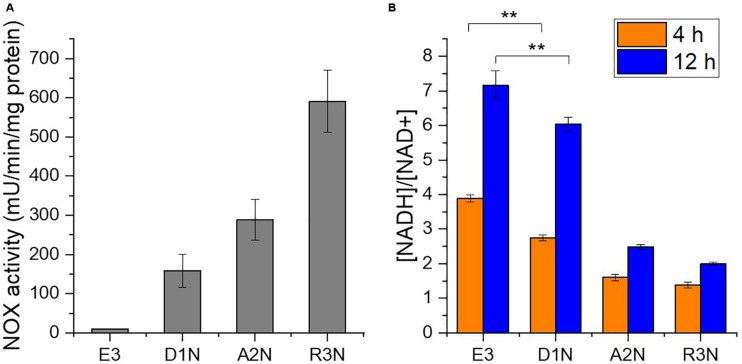
*In vitro* activity of NOX **(A)** and intracellular [NADH]/[NAD^+^] ratios **(B)**. D1N, A2N, and R3N indicate the NOX-expression strains with promoter *D1W12*p, *2A23*p and ermE*p, respectively. **, *P*-value < 0.01.

Three NOX-expression strains D1N, A2N, and R3N were constructed through integrating a single copy of NOX gene controlled by a weak promoter *D1W12*p, a medium promoter *2A23*p and the strong promoter *ermE*^∗^p (Wilkinson et al., 2002), respectively, into the chromosome. As a result, *in vitro* NOX activities in the three strains range from 150 to 580 mU/mg protein (Figure 2A), whereas E3 showed only trace NOX activity probably owing to cell debris containing NADH-consuming enzymes. NOX activities correlated well with the strength of promoters used.

We subsequently measured the intracellular [NADH]/[NAD^+^] ratio, which declined due to the expression of NOX both in the exponential phase (4 h) and in the stationary phase (12 h) of the cultivation (Figure 2B). The ratio determined during the exponential phase (4 h) should reflect the direct impact of expressing the NOX in *S. erythraea*. The [NADH]/[NAD^+^] ratios in the NOX-expression strain with the weak promoter *D1W12*p showed marked decrease compared with E3. The ratio in the strain with a stronger promoter *2A23*p decreased more significantly by 59 or 65% at 4 h or 12 h, respectively. Although the ratio in the strongest promoter *ermE*^∗^p were the lowest with a 64% decrease compared to E3 at 4 h, the difference between A2N and R3N which may reflect other factors, e.g., limited supply of oxygen or intracellular NADH. Nevertheless, the [NADH]/[NAD^+^] ratio at 12 h indicates a continuous NADH oxidation by NOX.

### The Expression of NOX Enhanced Erythromycin Production and Reduced Pigment Production

**FIGURE 3:**
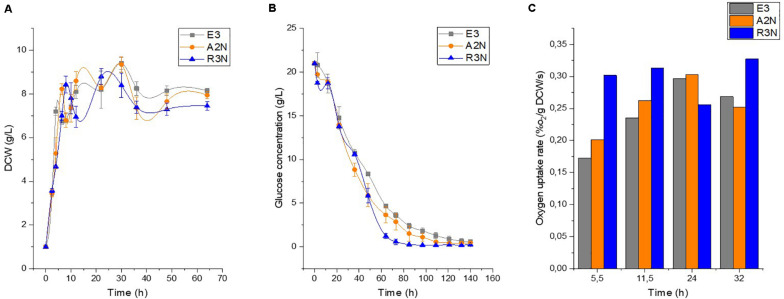
Physiological comparison between E3 and the NOX-expression strains regarding the growth, namely dry cell weight (DCW) **(A)**, the glucose consumption **(B)**, and the oxygen uptake rate **(C)**. Data are shown as mean ± standard deviation from at least three independent experiments for measuring growth and glucose concentration. Oxygen utilization was obtained from duplicate experiments.

Next, we conducted physiological characterization of the NOX-expression strains along with E3 in batch culture (Figure 3). The expression of NOX had a negative but moderate effect on the cellular growth rate and compared to E3. Besides, the expression of NOX exerted a negligible effect on the final formation of biomass and stimulated the glucose consumption rates. Figure 3A shows that at 10 h lysis of some cells occurred as a broad range of mRNA starts to degrade (Marcellin et al., 2013), and before this time point R3N showed the fastest glucose consumption rate, followed by A2N and E3. The difference in glucose consumption rates was maintained throughout the cultivation. This means that glycolysis in the NOX-expression strains was enhanced significantly, which increased NADH formation through glucose catabolism. The expression of NOX enhanced the specific oxygen uptake rate (r_O__2_) compared to E3. R3N consumed oxygen at the highest rate during the exponential growth phase compared to E3 and A2N. The r_O__2_ of R3N was comparatively stable over the cultivation process, while r_O__2_ of E3 or A2N increased steadily before 24 h. This implies that the oxygen supply for R3N might be limited under the standard experimental condition. For D1N, we did not observe a significant difference in physiology relative to E3 (data not shown).

**FIGURE 4:**
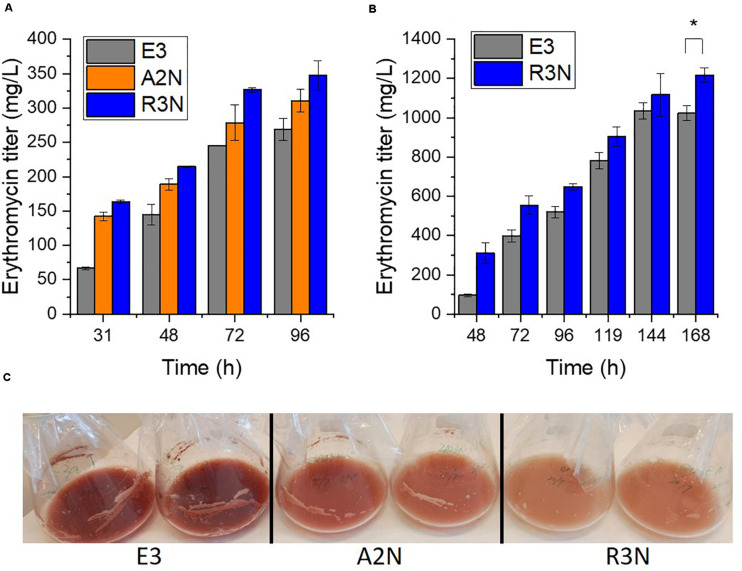
The expression of NOX redirected secondary metabolism. The NOX-expression strains produced more erythromycin in the minimal liquid medium **(A)** and the complex medium **(B)**, while the pigment production in the NOX-expression strains reduced dependent on the level of NOX expression in the minimal liquid medium **(C)**. Data are shown as mean ± standard deviation from at least three independent experiments for erythromycin determination. *, *P*-value < 0.05.

We then explored how the expression of NOX affected the secondary metabolism of *S. erythraea* (Figure 4). In general, the expression of NOX reduced reddish pigment (7-O-rahmnosyl flaviolin) production with increasing promoter strengths in liquid culture, while erythromycin production was enhanced as the expression of NOX increased (Figure 4C). Compared to E3, erythromycin production by A2N and R3N in the minimal liquid medium were increased by 15% and 29%, respectively. These data fit well with our previous study which showed that the anticipated increase in [NADH]/[NAD^+^] ratios after adding the inhibitor of NADH dehydrogenase, i.e., rotenone, exerted opposite effects on pigment and erythromycin production in *S. erythraea* (Li et al., 2020). Taken together, we have therefore demonstrated a negative correlation between the [NADH]/[NAD^+^] ratio and biosynthesis of erythromycin in *S. erythraea*, and a positive correlation between the redox ratios and the pigment production. We employed the complex medium closer to the medium used for the industrial production (Chen et al., 2017). Although there was no insignificant variance between the production of erythromycin by A2N and E3 (data not shown) R3N also exhibited 19% increase in erythromycin production relative to E3 (Figure 4B), which indicated that redox regulation could be a feasible strategy to stimulate the biosynthesis of erythromycin. On the other hand, changes of physiological parameters determined in this section were not linearly correlated with strength of NOX promoter.

### The Effect of Oxygen on the *in vivo* Activities of NOX

**FIGURE 5:**
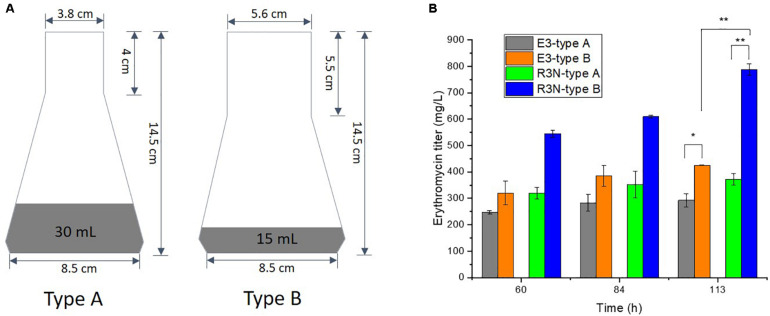
Effect of oxygen supply on the production of erythromycin by E3 and R3N. We used two types of shake flasks with different amounts of medium that is 30 mL for type A and 15 mL for type B **(A)**, resulting in differential erythromycin increase for E3 and R3N before and after increasing oxygen supply **(B)**. Data are shown as mean ± standard deviation from at least three independent experiments. *, *P*-value < 0.05; **, *P*-value < 0.01.

The slight decrease in [NADH]/[NAD^+^] ratios from A2N to R3N (Figure 2B) and the comparative constant r_O__2_ (Figure 3C) indicated that the oxygen transfer might affect functioning of NOX in R3N. We therefore increased the oxygen supply by changing setup for batch cultivation with the minimal liquid medium. Shake flasks with different sizes of bottleneck and charging with different volumes of culture were used for comparing the production of erythromycin (Figure 5). Type B shake flasks with less culture were expected to result in a higher oxygen transport rate to cells than type A (Auro et al., 1957; Henzler and Schedel, 1991). When both strains, i.e., R3N and E3 were cultivated in type A flasks, the production of erythromycin by R3N was increased by 25% compared to E3. When using type B shake flask, the production of erythromycin by E3 and R3N as well were stimulated compared to their respective cultivation in type A. E3 cultivated in type B showed a 46% increased production compared to type A, whereas the increase for R3N was 112%. Thus, under the standard cultivation in type A flasks, the expression of NOX in R3N improved the production of erythromycin by 25∼29% (Figure 4A and green vs. gray bars in Figure 5B), whereas the improvement was as high as 60% when cultivated in type B flasks (blue vs. orange bars in Figure 5B).

### A Comparison of Redox and Energy Perturbation in *S. erythraea*

Under aerobic condition, NADH oxidation is interconnected tightly to ATP generation and our recent study showed that higher intracellular [ATP]/[ADP] ratios stimulated the biosynthesis of erythromycin (Li et al., 2020). The current study showed the positive effects exerted by lower [NADH]/[NAD^+^] ratios or enhanced oxygen supply on the production of erythromycin (Figures 4, 5). However, the exact role of oxygen in the biosynthesis of erythromycin is still not fully understood. We then compared the intracellular cofactor levels of R3N and E3H (E3:F1F0ATPase), which overexpresses the native F_1_F_0_-ATPase (Li et al., 2020). The two engineered strains have approximately the same increase level of erythromycin titer.

**FIGURE 6:**
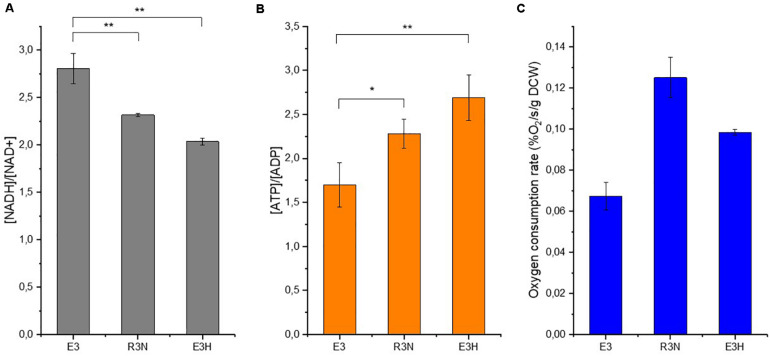
Intracellular cofactor ratios, [NADH]/[NAD^+^] **(A)** and [ATP]/[ADP] **(B)**, oxygen consumption rate at the same time **(C)** in E3, R3N and E3:F1F0ATPase (E3H). Data are shown as mean ± standard deviation from three independent experiments. Asterisks indicate the significance of variance. *, *P*-value < 0.05; **, *P*-value < 0.01.

Samples from cell cultures in minimal liquid medium were withdrawn after 4 h for determination of intracellular cofactors and oxygen uptake rate. E3H exhibited the lowest [NADH]/[NAD^+^] ratio but the highest [ATP]/[ADP] ratio among the strains (Figure 6). The [ATP]/[ADP] ratio in R3N was increased moderately relative to E3. When the reduced biomass formation for E3H (Li et al., 2020) and the comparable biomass formation for R3N relative to E3 in the minimal liquid medium were taken into account, the E3H cells showed the strongest capability to produce erythromycin. This fits the established correlations between [NADH]/[NAD^+^] or [ATP]/[ADP] ratios and the biosynthesis of erythromycin. R3N and E3H both exhibited faster oxygen consumption rate relative to E3, while R3N consumed oxygen 26% faster than E3H (Figure 6C). The difference in [NADH]/[NAD^+^] ratios between R3N and E3H was most likely due to the different amounts of glucose assimilated by the two strains, as evidenced by the enhanced glucose uptake for R3N and the repressed glucose uptake for E3H, both relative to E3 (Supplementary Figure S3).

### Transcriptional Changes Exerted by the Expression of NOX in *S. erythraea*

Since most physiological alterations originate at the transcriptional level, we analyzed the genome-wide transcription response to the redox perturbation with samples withdrawn in early exponential phase (4 h) for E3 and the NOX-expression strains. When cells grow in the early exponential phase, few limitations are imposed on the cell, which facilitates uncovering the direct effects of the changed redox status. Furthermore, in the early exponential phase, genes in erythromycin BGC show relatively high transcription values relative to the stationary phase (Li et al., 2013; Karnicar et al., 2016).

**FIGURE 7:**
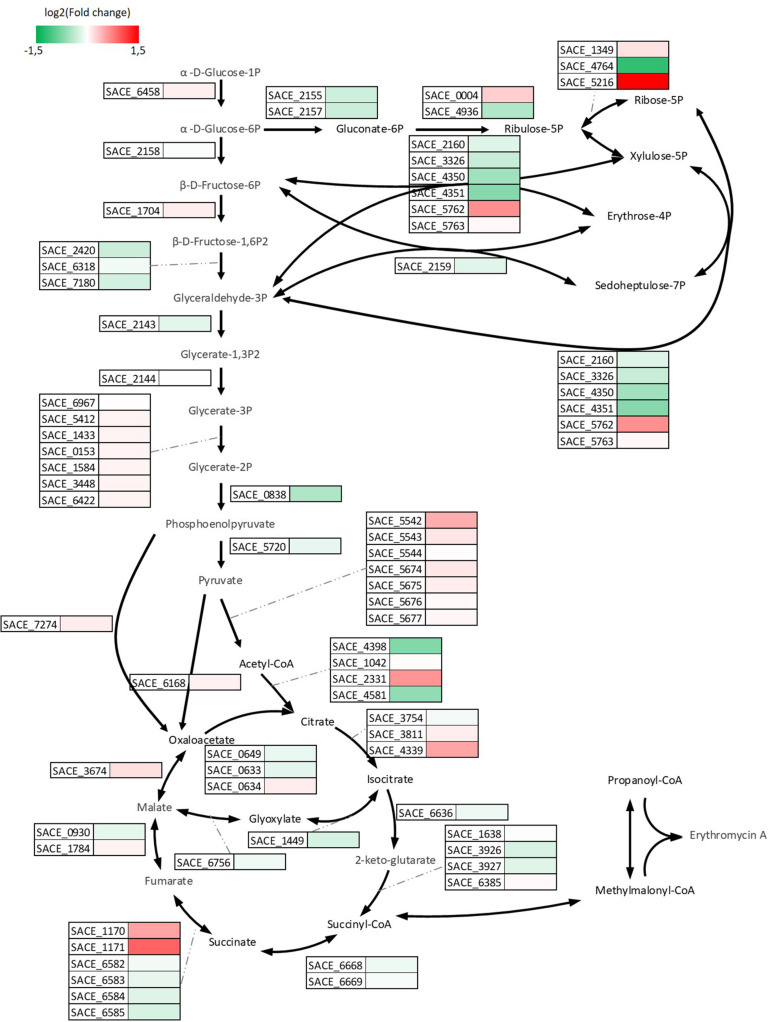
Visualization of differences in central carbon metabolism at transcription level. Rectangular with color beside the black arrows represent the relative transcription level of genes responsible for the reactions in A2N compared to E3.

Differentially expressed genes analysis revealed that 88 genes were induced and 14 genes were repressed significantly in A2N relative to E3. Genes responsible for (i) DNA repair and replication, (ii) inorganic ion transport and metabolism, and (iii) carbohydrate metabolism and transport were induced in terms of cluster orthologous group (COG) functional categories of DEGs (Supplementary Table S2). About two thirds of genes repressed in A2N belonged to category of carbohydrate metabolism and transport, implying that *S. erythraea* rerouted carbon flux in response to the redox perturbation. As shown in Figure 7, the transcription of genes among the central carbon metabolism was altered. Most genes involved in glycolytic pathway were induced moderately, as an increased glucose uptake in A2N resulted in the enhanced formation of NADH through the glycolytic pathway. Genes coding for the predominant succinate dehydrogenase (Sdh) complex, i.e., SACE_6582∼6585, were repressed marginally, whereas the other Sdh complex encoded by SACE_1170/1171 was expressed lowly at the transcriptional level, although SACE_1170/1171 presented significant up-regulation in A2N. SACE_0302 codes for a catalase and showed the highest log2 (fold change), 4.6 and 5.2, in both A2N and R3N. The induction of a catalase implied that the NOX-expression strains also expressed higher capability to alleviate the oxidative stress that is reflected in reactive oxygen species (ROSs). More than a quarter of DEGs coding for transporters or transferases were associated with the transport of carbohydrates, and the transfer of DNA or chemical groups among molecules. For instance, catabolism of poly- or oligo-saccharide and arabinose were up-regulated significantly while metabolism of fructose and sugar phosphate was down-regulated.

We also investigated genes with descending tendency at the transcriptional level in E3, A2N, and R3N to elucidate the cellular response, which counteracts the redox perturbation (Supplementary Table S3). Genes involved in (i) transcription, (ii) energy production and conversion, and (iii) carbohydrate metabolism and transport responded most actively. As the regulatory networks of erythromycin BGC remain elusive, the genes encoding transcriptional regulators with descending transcriptional tendency across strains hints at the underlying regulation mechanism of the BGC. Another key gene with descending tendency was SACE_0142 (*cydB*) which codes for *bd* type terminal oxidase at the ETC. The slightly transcriptional change of genes coding for succinate dehydrogenase indicated a comparable amount of electrons transported through ETC in A2N compared to E3.

### Links Between Redox Status and the Biosynthesis of the Reddish Pigment or Erythromycin

In the present work we demonstrated the correlation between [NADH]/[NAD^+^] ratios and biosynthesis of pigment or erythromycin. Nevertheless, it is still unclear how the redox status regulates the biosynthesis of pigment or erythromycin at the molecular level. Since biosynthesis of secondary metabolites are rather complex processes associated with the supply of precursors and cofactors from primary metabolism, we here only focus on the synthesis steps starting from the assembly of primary precursors.

The synthesis of the reddish pigment, i.e., 7-O-rahmnosyl flaviolin starts with chain extension of malonyl-CoA, which is catalyzed by acetyl-CoA carboxylase (Cortés et al., 2002). The process is under the positive transcriptional regulation by DasR (Liao et al., 2015). Table 3 lists genes involved in the biosynthesis of the pigment. Most of the genes in Table 3 exhibited relatively low transcription values, while SACE_0500 (*dasR*) showed a much higher transcription level. It appears that even in the early exponential phase *dasR* was repressed with increasing expression of NOX, which could lead to a decrease in pigment production in the NOX-expression strains at transcription level (Liao et al., 2015). Furthermore SACE_3400 and SACE_6509 coding for acetyl-CoA carboxylase were repressed gradually in the NOX-expression strains. The repression of SACE_3400 and SACE_6509 could reduce the accumulation of malonyl-CoA from acetyl-CoA. The transcriptional changes of these genes therefore accounted for the decrease of pigment production in the NOX-expression strains. Malonyl-CoA cannot be converted into propionyl-CoA, one of the precursors of erythromycin, probably due to the lack of enzymes to catalyze the conversion from malonyl-CoA to 3-hydroxy-propionyl-CoA (Cortés et al., 2002). Therefore, the repressed conversion from acetyl-CoA to malonyl-CoA could drive more carbon flux to succinyl-CoA through the TCA cycle. As the TCA cycle is the main source of methylmalonyl-CoA, which is another precursor of erythromycin, the biosynthesis of erythromycin could benefit from the repression of acetyl-CoA carboxylase.

**TABLE 3:** Transcription level of genes involved in the biosynthesis of pigment (7-O-rahmnosyl flaviolin).

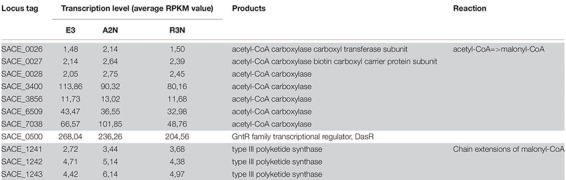

**FIGURE 8:**
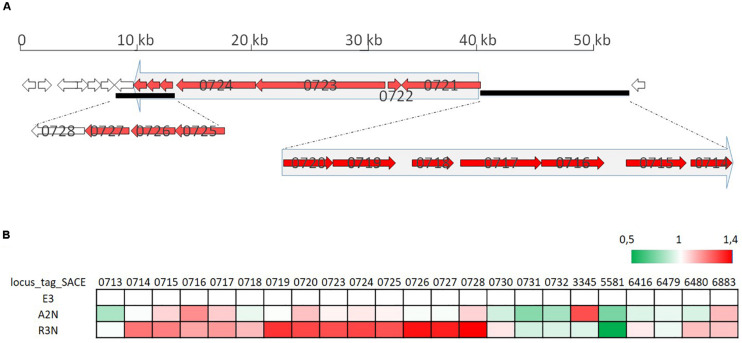
Organization of the erythromycin biosynthesis gene cluster **(A)** and its transcription profiles **(B)**. Small arrows in **(A)** indicate coding regions of the erythromycin biosynthesis gene cluster and numbers on arrows are gene locus tags. Two big arrows in light gray consisting of several genes in red indicate the genes are co-transcribed. **(B)** shows relative transcription of genes in the cluster for E3, A2N, and R3N.

With respect to the biosynthesis of erythromycin, within the same strains all the genes in the erythromycin BGC had higher transcription levels compared to pigment genes. Part of the gene cluster (SACE_0714-0728) was induced slightly in A2N, but significantly by 25∼30% in R3N (Figure 8). The genes (SACE_6480/6883) responsible for metabolism of one of erythromycin precursors, dTDP-sugar, were also induced. Transcripts of SACE_0714∼0728 are divided into two parts (Reeves et al., 1999) and their transcription are both under directly positive regulation of BldD (Chng et al., 2008). However, the transcription of *bldD* showed merely 10% increase in both A2N and R3N, which could not completely account for the upregulation of erythromycin BGC.

A previous study has proved that c-di-GMP was able to activate erythromycin BGC transcription by strengthening the binding of BldD to promoters of erythromycin BGC and E3 had higher concentrations of intracellular c-di-GMP compared to the wild type of *S. erythraea* (Xu et al., 2019). In *S. erythraea*, the diguanylate cyclase catalyzes the synthesis of c-di-GMP with guanosine-5′-triphosphate (GTP) as the sole substrate and the phosphodiesterase functions to hydrolyze c-di-GMP. However, transcription of all genes coding for diguanylate cyclase or phosphodiesterase in NOX-expression strains appeared comparable to that in E3 (Supplementary Table S4), which implied that the NADH somehow functioned as an inhibitor of diguanylate cyclase.

Therefore, we investigated if NADH exerted any effects on the activity of diguanylate cyclase. The diguanylate cyclase catalyzes the conversion from GTP to c-di-GMP and diphosphate. A whole cell-free extract was prepared with cells grown in exponential phase to determine the activity of diguanylate cyclase, which was reflected in the rate of formation of c-di-GMP with GTP as the only substrate. Table 4 shows that c-di-GMP formation was inhibited by 9% with 0.2 mM NADH addition relative to the results without NADH. The inhibition increased to 15% when introducing 1 or 5 mM NADH into the reaction system. The non-linear correlation between the inhibition of c-di-GMP formation and the level of NADH indicated that NADH may interact directly with diguanylate cyclase, as previously observed for the inhibition of adenylate cyclase by NADH (Nair and Patel, 1991). The expression of NOX could alleviate the inhibition of diguanylate cyclase activity by NADH, and thus the transcription of erythromycin BGC could be enhanced probably because higher concentration of c-di-GMP in the NOX-expression strains enhanced the binding between the positive regulator BldD and erythromycin BGC (Chng et al., 2008; Xu et al., 2019).

**TABLE 4:** Effect of NADH on the activity of diguanylate cyclase.

**NADH addition (mM)**	c-di-GMP formation (nmol/μg protein*/20 min)**	*P*-value***
0	0.077 ± 0.002	–
0.2	0.071 ± 0.001	0.0202
1	0.066 ± 0.002	0.0031
5	0.066 ± 0.001	0.0024

* The concentration of total protein in the whole cell extract was taken into account. ** Data are shown as mean ± standard deviation from three independent experiments. *** Significance test was performed by calculating the *P*-value of c-di-GMP formation with NADH addition relative to the condition without NADH.

## Discussion

In previous studies regarding engineering *S. erythraea* only defined promoters from *S. erythraea* or limited numbers of promoters originated from streptomycetes can be employed (Chen et al., 2008; Xu et al., 2019). The library in this study was perhaps not perfect, but broad enough for the required expression of *nox* gene in *S. erythraea*. It is indeed likely that with selecting more colonies exhibiting the GUS activity, it would be possible to have promoters stronger than *ermE*^∗^p. In fact, when we performed the primary screening using the GUS activity on agar plates, several tiny colonies each with a very strong blue halo were collected (data not shown). Unfortunately, these colonies cannot grow well in liquid medium so that we did not compare their strength quantitatively, but it is likely that the strength of these promoters was higher than the *ermE*^∗^p, as strains with *ermE*^∗^p-gusA can grow well on both agar plates and in liquid medium. The current study proposed a readily available strategy to construct a SPL based on sequence alignments and nucleotide randomization. The success in designing our SPL suggests a semi-*de novo* method to create genetic tools in a wide range of microorganisms.

Under *in vitro* conditions with sufficient substrates, the results of NOX assay reflected the amount of NOX expressed by cells. The non-linear correlation between physiological changes and NOX activities indicated that additional limitation occurred in R3N (Figure 2). The oxygen uptake rate appeared to limit fully functioning of NOX in R3N under the experimental condition, which was evidenced by the disproportionate increase of r_O__2_ among E3, A2N and R3N, relative to the promoter strength (Figure 3). In addition, the higher production of erythromycin in R3N under oxygen-enriched conditions reflected the oxygen limitation for R3N in the current standard cultivation and the important role of oxygen in erythromycin production (Figure 5). Streptomycetes and *S. erythraea* predominantly use oxygen to accept electrons (Hong et al., 2016). The expression of NOX uncouples both the oxygen consumption and NADH oxidization from ATP production. As a consequence, the majority of the increased part of oxygen consumption in the NOX-expression strains relative to the control strain in Figure 6C was used by NOX for redox regulation rather than by ETC for ATP generation, as evidenced by the moderate increase of ATP level in R3N compared to E3 (Figure 6B). The moderate increase of ATP level induced by the expression of NOX were also observed in *S. cerevisiae* and *E. coli* (Hou et al., 2009; Holm et al., 2010). This indicated that the NOX-expression strains were in urgent demand of redox regulation instead of energy generation with respect to the role of oxygen consumption.

The expression of NOX led to down-regulation of *bd* terminal oxidase by the transcriptional regulation of Rex (Brekasis and Paget, 2003; Supplementary Table S3), while the genes encoding *bc_1_-aa_3_* terminal oxidase exhibited slight up-regulation at the transcriptional level in comparison to E3. Given the different efficiency in generating the electrochemical gradient of protons coupled to the electron transport: *bc_1_-aa_3_* (6H^+^/2e^–^) and *bd* (2H^+^/2e^–^) (Fujimoto et al., 2016), the rerouting of electron transport indicates the possibility that the NOX-expression strains could translocate a comparable amount of H^+^ relative to E3 across the membrane at the expense of transporting less electrons. The transcriptional change of ETC genes implied that excessive NADH existed in E3 relative to the least demand of reducing power for generating enough energy. This was evidenced by the constant transcriptional levels of genes coding for the NADH dehydrogenase complex in R3N, but generated a moderately larger amount of ATP compared to E3 (Figure 6). The excessive NADH in E3 could also account for the enhancement of erythromycin titer when oxygen supply increased (Figure 5). The NADH distribution among NOX and NADH dehydrogenases can also be explained by their different affinities for NADH. NOX from *Streptococcus* exhibits a lower *K*_m_ value, 25 μM for NADH (Higuchi et al., 1993) while the *K*_m_ value is 100 μM for membrane-bounded NADH dehydrogenase (Vinogradov, 2008). Concomitantly, as NOX oxidized more NADH to NAD^+^, the NOX-expression strain produced less ATP with consuming more oxygen compared to E3H (Figure 6C). *S. cerevisiae* showed a similar phenomenon that the expression of NOX led to a lower energetic efficiency based on oxygen consumption (Hou et al., 2009). Taken together, the comparison among the three strains emphasized the redox regulation as the prior role of oxygen consumption in *S. erythraea*. Further process optimization to reduce intracellular redox status for industrial cultivation of E3 is worthwhile to improve erythromycin titer on a larger scale.

**FIGURE 9:**
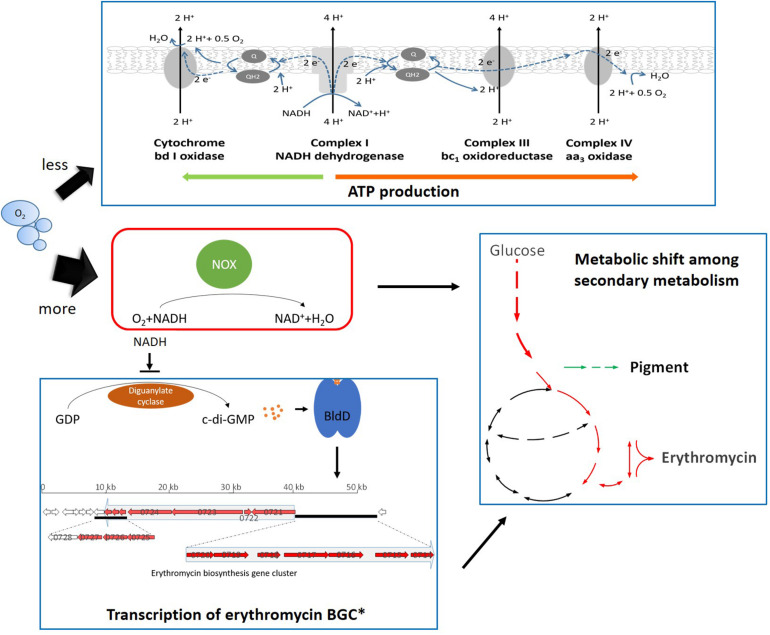
Schematic representation of the cellular response toward the expression of NOX in *S. erythraea*. Black thin arrows refer to positive regulation; the thin arrows ended by a perpendicular short line refer to negative regulation. Green arrows beside *Pigment* indicates down-regulation, while red arrows beside *Erythromycin* indicates up-regulation. * refers to 2 previous studies (Chng et al., 2008; Xu et al., 2019).

It was widely observed that the alteration of NADH availability or redox status could lead to metabolic re-distribution among primary metabolism in order to maintain the redox balance (De Felipe et al., 1998; Berrios-Rivera et al., 2004). In *S. erythraea*, the stimulated uptake of glucose and transcriptional changes showed that the NOX-expression strains shifted the cellular metabolism to achieve more NADH formation (Figures 3, 7). In addition, a most interesting metabolic shift among secondary metabolism occurred which reflected in the reverse response of the biosynthesis of erythromycin or the reddish pigment (Figure 4). Acetyl-CoA plays vital roles in branching metabolism. Via carboxylation or the TCA cycle, acetyl-CoA is converted to malonyl-CoA or (2S)-methylmalonyl-CoA, which is the necessary precursor for the biosynthesis of the reddish pigment and erythromycin, respectively (Li et al., 2020). Furthermore, the TCA cycle is the main source of (2S)-methylmalonyl-CoA in *S. erythraea* (Hong et al., 2016; Xu et al., 2018). The biosynthesis of erythromycin generates twice as much NADH compared to pigment production (Li et al., 2020). In *S. cerevisiae*, the NOX-expression strain also compensates for the decreased intracellular NADH availability by promoting NAD^+^-dependent reactions (Heux et al., 2006). Therefore the secondary metabolic change in *S. erythraea* was another example of the shift toward more NADH (Figure 9).

When it comes to genetic mechanisms to trigger the physiological response toward redox perturbation, the present work established a link between the cellular redox signal and transcriptional regulation of the erythromycin BGC. The transcription of erythromycin BGC is activated by the binding of a positive regulator, BldD (Chng et al., 2008). Secondary messenger c-di-GMP has been shown to strengthen BldD’s binding to erythromycin BGC and E3 had higher concentrations of intracellular c-di-GMP compared to the wild type of *S. erythraea* (Xu et al., 2019). Although more research is worth trying and essential, our work indicated that higher NADH in the cells can inhibit the activity of diguanylate cyclase, which may result in lower accumulation of c-di-GMP and then decreasing the biosynthesis of erythromycin (Figure 9). Therefore, the expression of NOX or the sufficient supply of oxygen could benefit the accumulation of c-di-GMP which in turn leads to enhanced erythromycin production.

## Data Availability Statement

Raw reads of RNA sequencing used in the present study can be obtained from the NCBI Gene Expression Omnibus (accession number: GSE144067).

## Author Contributions

All authors read and approved the manuscript, contributed significantly to the work, and conceived the project. XL and PJ designed the experiments and analyzed the results. XL wrote the manuscript with the help of JC and PJ. XL performed the experiments and supported by JC and PJ.

## Conflict of Interest

The authors declare that the research was conducted in the absence of any commercial or financial relationships that could be construed as a potential conflict of interest.

## Supplementary Material

FIGURE S1 Search for putative 16s rRNA promoters in *S. erythraea*.

FIGURE S2 Coding sequence of NADH oxidase after codon optimization.

FIGURE S3 Concentration of residual glucose for E3 and E3:F1F0ATPase (E3H) in 1 L fermenters with 600 mL minimal liquid medium.

TABLE S1 Sequences of promoters.

TABLE S2 Differentially expressed genes in A2N relative to E3.

TABLE S3 Genes with descending tendency at the transcription level in E3, A2N, and R3N.

TABLE S4 Transcription levels of genes coding for diguanylate cyclase and phosphodiesterase in *S. erythraea*.
