# Supplementary material for: The Expression of NOX From Synthetic Promoters Reveals an Important Role of the Redox Status in Regulating Secondary Metabolism of Saccharopolyspora erythraea
Source: Front Bioeng Biotechnol. 2020 Jul 17;8:818. doi: 10.3389/fbioe.2020.00818 (PMC7379104; doi:10.3389/fbioe.2020.00818)
Supplement: FIGURE S1 — Search for putative 16s rRNA promoters in S. erythraea. [file Data_Sheet_1.docx]

**The expression of NOX from synthetic promoters reveals an important role of the redox status in regulating secondary metabolism of *Saccharopolyspora erythraea***

**Xiaobo Li^a,b^, Ju Chu^a#^, and Peter Ruhdal Jensen^b#^**

a State Key Laboratory of Bioreactor Engineering, East China University of Science and Technology, 130 Meilong Road, Shanghai 200237, People’s Republic of China

b National Food Institute, Technical University of Denmark, Kemitorvet, Building 201, 2800 Kongens Lyngby, Denmark

#Corresponding authors:

Ju Chu: juchu@ecust.edu.cn; Tel. (+86) 021-64253021

Peter Ruhdal Jensen: perj@food.dtu.dk; Tel. (+45) 20855601

Contents

[Figure S1. Search for putative 16s rRNA promoters in *S. erythraea*.. 2](#_Toc38891621)

[Figure S2. Coding sequence of NADH oxidase after codon optimization. 3](#_Toc38891622)

[Figure S3. Concentration of residual glucose for E3 and E3::F1F0ATPase (E3H) in 1 L fermenters with 600 mL minimal liquid medium. 3](#_Toc38891623)


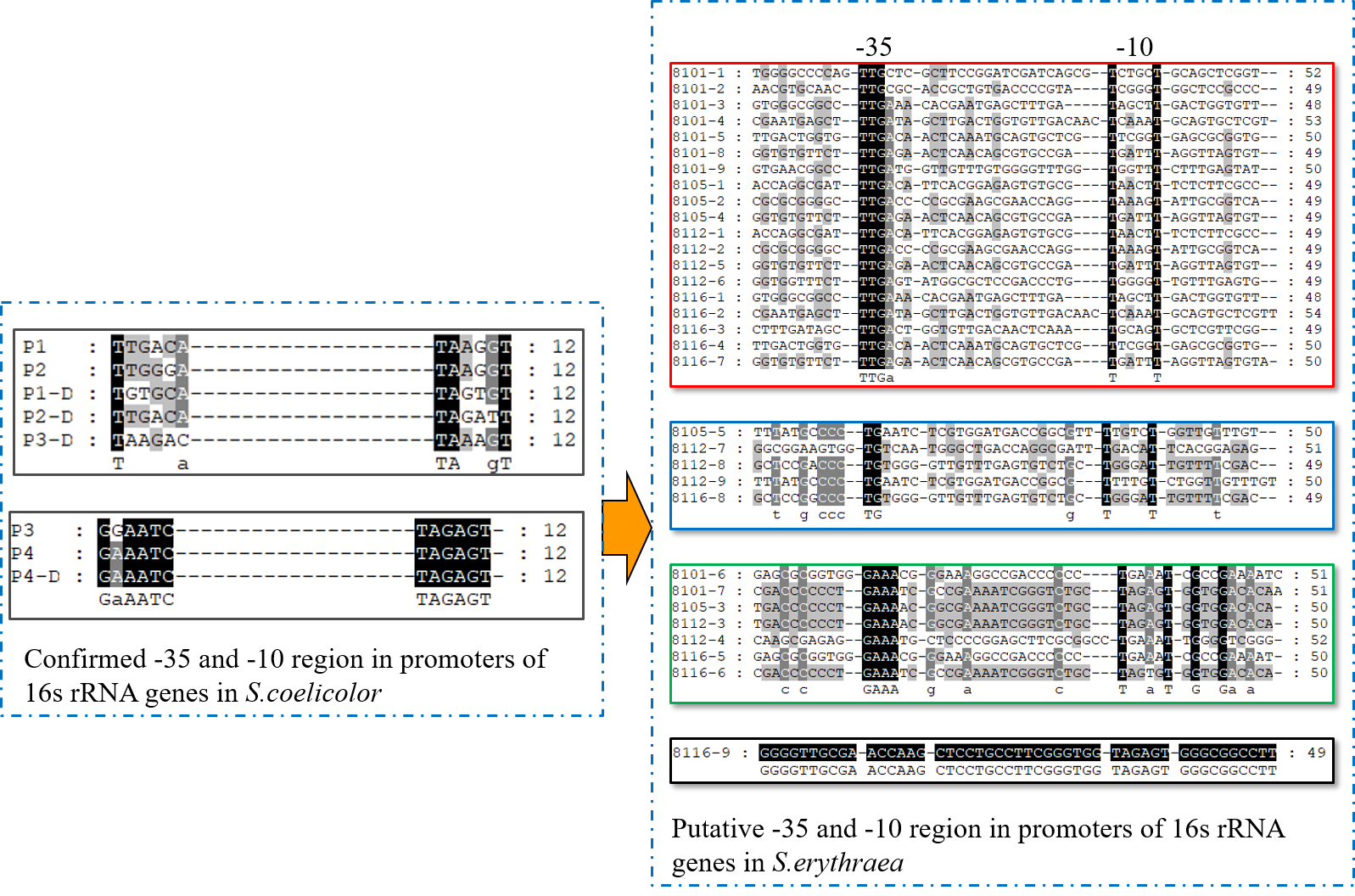


# Figure S1. Search for putative 16s rRNA promoters in *S. erythraea*. In the left panel -35 and -10 regions of 16s rRNA genes in *S. coelicolor* were listed and summarized with black and grey background. P1/P2/P3/P4 are the promoter names in *S .coelicolor*. The consensus sequences were employed to identify the putative -35 and -10 regions in *S. erythraea* by searching for similar sequences upstream of the coding sequences of 16s rRNA genes in *S. erythraea* in the right panel. 8101/8105/8112/8116 are locus tags of 16s rRNA genes in *S. erythraea*. Numbers at the end of sequences indicate their length. The upstream, -35 region, spacer, -10 region and the downstream were separated from each other by “-” gaps.

5’-ATGTCCAAGATCGTCGTCGTCGGCGCCAACCACGCCGGCACCGCCTGCATCAACACCATGCTGGACAACTTCGGCAACGAGAACGAGATCGTCGTCTTCGACCAGAACTCCAACATCTCCTTCCTGGGCTGCGGCATGGCCCTGTGGATCGGCGAGCAGATCGACGGCGCCGAGGGCCTGTTCTACTCCGACAAGGAGAAGCTGGAGGCCAAGGGCGCCAAGGTCTACATGAACTCCCCGGTCCTGTCCATCGACTACGACAACAAGGTCGTCACCGCCGAGGTCGAGGGCAAGGAGCACAAGGAGTCCTACGAGAAGCTGATCTTCGCCACCGGCTCCACCCCGATCCTGCCGCCGATCGAGGGCGTCGAGATCGTCAAGGGCAACCGCGAGTTCAAGGCCACCCTGGAGAACGTCCAGTTCGTCAAGCTGTACCAGAACGCCGAGGAGGTCATCAACAAGCTGTCCGACAAGTCCCAGCACCTGGACCGCATCGCCGTCGTCGGCGGCGGCTACATCGGCGTCGAGCTGGCCGAGGCCTTCGAGCGCCTGGGCAAGGAGGTCGTCCTGGTCGACATCGTCGACACCGTCCTGAACGGCTACTACGACAAGGACTTCACCCAGATGATGGCCAAGAACCTGGAGGACCACAACATCCGCCTGGCCCTGGGCCAGACCGTCAAGGCCATCGAGGGCGACGGCAAGGTCGAGCGCCTGATCACCGACAAGGAGTCCTTCGACGTCGACATGGTCATCCTGGCCGTCGGCTTCCGCCCGAACACCGCCCTGGCCGACGGCAAGATCGAGCTGTTCCGCAACGGCGCCTTCCTGGTCGACAAGAAGCAGGAGACCTCCATCCCGGGCGTCTACGCCGTCGGCGACTGCGCCACCGTCTACGACAACGCCCGCAAGGACACCTCCTACATCGCCCTGGCCTCCAACGCCGTCCGCACCGGCATCGTCGGCGCCTACAACGCCTGCGGCCACGAGCTGGAGGGCATCGGCGTCCAGGGCTCCAACGGCATCTCCATCTACGGCCTGCACATGGTCTCCACCGGCCTGACCCTGGAGAAGGCCAAGGCCGCCGGCTACAACGCCACCGAGACCGGCTTCAACGACCTGCAGAAGCCGGAGTTCATGAAGCACGACAACCACGAGGTCGCCATCAAGATCGTCTTCGACAAGGACTCCCGCGAGATCCTGGGCGCCCAGATGGTCTCCCACGACATCGCCATCTCCATGGGCATCCACATGTTCTCCCTGGCCATCCAGGAGCACGTCACCATCGACAAGCTGGCCCTGACCGACCTGTTCTTCCTGCCGCACTTCAACAAGCCGTACAACTACATCACCATGGCCGCCCTGACCGCCGAGAAGTGA -3’

# Figure S2. Coding sequence of NADH oxidase after codon optimization.

# Figure S3. Concentration of residual glucose for E3 and E3::F1F0ATPase (E3H) in 1 L fermenters with 600 mL minimal liquid medium.
